# Impact of articulating laparoscopic instrument–assisted gastrectomy with D2 lymphadenectomy on perioperative and oncologic outcomes compared with conventional laparoscopy: a propensity score matching analysis

**DOI:** 10.1007/s00464-025-11976-y

**Published:** 2025-07-10

**Authors:** Seohee Choi, Takahiro Kinoshita, Kazutaka Obama, Katsunobu Sakurai, Naoshi Kubo, Naruhiko Ikoma, Ali Guner, Hyoung-Il Kim

**Affiliations:** 1 Department of Surgery, National Health Insurance Service Ilsan Hospital, Goyang, Republic of Korea; 2 Gastric Surgery Division, National Cancer Center Hospital East, Chiba, Japan; 3 Department of Surgery, Graduate School of Medicine, Kyoto University, Kyoto, Japan; 4 Department of Gastroenterological Surgery, Osaka City General Hospital, Osaka, Japan; 5 Department of Surgical Oncology, The University of Texas MD Anderson Cancer Center, Houston, TX USA; 6 Department of Surgery, Faculty of Medicine, Karadeniz Technical University, Trabzon, Turkey; 7 Department of Surgery, Yonsei University College of Medicine, Seoul, Republic of Korea; 8 Gastric Cancer Center, Yonsei Cancer Center, Yonsei University Health System, Seoul, Republic of Korea

**Keywords:** Articulating laparoscopic instrument, Gastrectomy, Laparoscopy, D2 lymphadenectomy, Gastric cancer

## Abstract

**Background:**

Articulating laparoscopic instruments (ALIs) have been developed to overcome the limited dexterity afforded by conventional laparoscopic instruments (CLIs). This study aimed to compare the postoperative and oncologic outcomes of patients who underwent laparoscopic gastrectomy with D2 lymphadenectomy for gastric cancer using CLIs versus ALIs.

**Methods:**

This retrospective study included 138 patients who underwent laparoscopic gastrectomy with D2 dissection for gastric cancer at a single institution from January 2018 to January 2024. Propensity score matching analysis was performed to minimize selection bias and compare surgical outcomes.

**Results:**

After matching, 39 patients were included in each group. The ALI group showed significantly faster postoperative recovery, with a shorter hospital stay (4.0 [3.0–5.0] days vs. 5.0 [4.0–7.0] days, p = 0.001) and quicker time to first flatus (2.0 [2.0–3.0] days vs. 3.0 [2.0–3.0] days, p = 0.004). Although the ALI group had a shorter operative time and lower estimated blood loss, these differences were not statistically significant (p = 0.202 and p = 0.634, respectively). Complication rates, including major complications, were similar between the two groups. Long-term oncologic outcomes, including overall survival and recurrence-free survival, did not differ significantly between the groups (p = 0.622 and p = 0.756, respectively).

**Conclusion:**

The use of ALIs in laparoscopic gastrectomy with D2 lymphadenectomy was associated with improved short-term perioperative outcomes without compromising long-term oncologic safety. These findings suggest that ALIs may enhance surgical efficiency and postoperative recovery in gastric cancer surgery.

**Graphical abstract:**

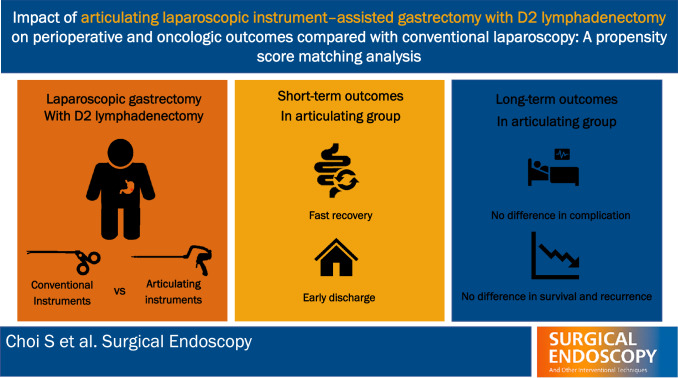

Laparoscopic gastrectomy has emerged as a minimally invasive surgical option for the treatment of early gastric cancer [1–3] and is increasingly used to treat advanced gastric cancer, offering benefits such as reduced postoperative pain, shorter hospital stays, and faster recovery compared with open surgery [4, 5]. However, the complexity of certain procedures, such as D2 lymph node dissection, could challenge the precision and flexibility of conventional laparoscopic instruments (CLIs) [6]. In response to these limitations, articulating laparoscopic instruments (ALIs) were developed as an alternative to CLIs, providing better access to difficult-to-reach areas during surgery due to their enhanced maneuverability.

Previous studies have primarily focused on the short-term outcomes of laparoscopic surgery using ALIs, reporting favorable perioperative results, including lower complication rates and quicker recovery times [7–9]. Furthermore, ALIs may reduce intraoperative stress and improve surgical precision, leading to better short-term recovery. However, despite the theoretical advantages, evidence comparing CLIs and ALIs in the context of oncological outcomes remains limited.

The purpose of this study was to compare the perioperative and oncologic outcomes of patients undergoing laparoscopic gastrectomy with D2 lymphadenectomy using either CLIs or ALIs.

## Methods

### Study design and patients

This retrospective study included a total of 78 patients matched from an initial cohort of 138 patients who underwent laparoscopic gastrectomy with D2 lymphadenectomy at Severance Hospital, Korea, between January 2018 and January 2024. Propensity score matching was conducted to account for potential confounding variables, using sex, age, American Society of Anesthesiologists (ASA) physical status, extent of gastrectomy, tumor stage, and body mass index (BMI) as matching criteria. After matching, the CLI group and the ALI group each included 39 patients. Patients with distant metastasis and those who received neoadjuvant chemotherapy were excluded from the study.

The study protocol was approved by the Institutional Review Board of Severance Hospital, Yonsei University College of Medicine (4-2024-1553).

### Surgical procedure

**Fig. 1:**
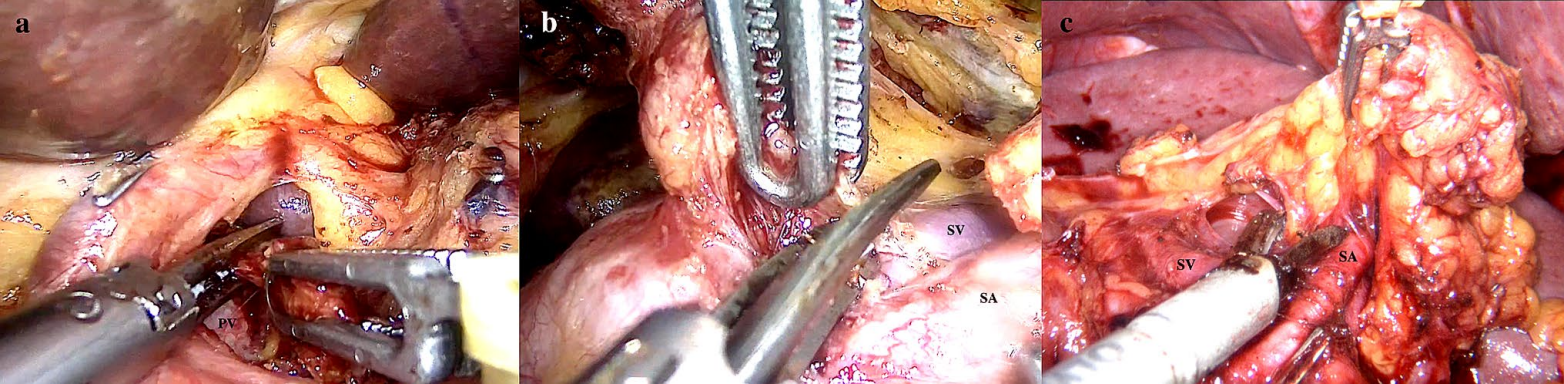
An intraoperative view of the articulating laparoscopic instruments (ALIs) in D2 lymphadenectomy. **a** Dissection of lymph node station No. 12a (along the proper hepatic artery); **b** dissection of the 11p area lymph node (along the proximal splenic artery); **c** dissection of the 10 area lymph node (splenic hilar region). *PV* portal vein; *SA* splenic artery; *SV* splenic vein

All surgeries were performed by an experienced surgeon specializing in laparoscopic gastric surgery. Patients in the CLI group underwent surgery using standard laparoscopic instruments, whereas those in the ALI group underwent surgery using ALIs, specifically ArtiSential fenestrated forceps (AUF01L, LivsMed, Seongnam, Korea). Laparoscopic gastrectomy with D2 lymphadenectomy was performed in both the CLI and ALI groups, following the guidelines of the Korean Gastric Cancer Association and the Japanese Gastric Cancer Association [10, 11]. In the ALI group, articulating instruments were used for lymph node dissection, particularly in lymph node stations 12a (proper hepatic artery), 11p (proximal splenic artery), and 10 (splenic hilum) (Fig. 1). The extent of gastrectomy was determined based on the tumor location and disease extent. The reconstruction method—Billroth I (gastroduodenostomy), Billroth II (gastrojejunostomy), Roux-en-Y gastrojejunostomy, or the double-flap technique—was chosen depending on the extent of the resection and the surgeon’s preference.

### Perioperative and postoperative management

Perioperative parameters included the operative time, the estimated intraoperative blood loss, and the need for blood transfusion. Postoperative outcomes included the duration of hospitalization, the time to first flatus, and the occurrence of complications, which were classified according to the Clavien–Dindo system [12]. Major complications were defined as grade III or higher. All patients followed a standardized postoperative care regimen, including early mobilization and the introduction of oral intake beginning with sips of water on the first postoperative day.

### Survival outcome measures

The primary outcomes were overall survival (OS) and recurrence-free survival (RFS). OS was defined as the time from surgery until the last follow-up or death from any cause. RFS was defined as the time from surgery until the first recurrence from any cause. Recurrence sites were documented for patients who experienced recurrence during the follow-up period. Follow-up was limited to a maximum of 36 months to ensure consistency among participants.

### Statistical analysis

Propensity score matching was performed to minimize selection bias and balance baseline characteristics between the two groups. The ALI group was defined as the treatment group, whereas the CLI group was defined as the control group. Propensity scores were calculated using a logistic regression model based on the following covariates: age, sex, extent of gastrectomy, ASA score, BMI, and stage. A nearest-neighbor matching algorithm with a caliper width of 0.2 was used to match patients in a 1:1 ratio. Categorical variables were presented as counts and percentages and were compared between groups using the chi-square test or Fisher’s exact test. Continuous variables were expressed as medians and interquartile ranges and compared using the Mann–Whitney U test. Kaplan–Meier survival curves were generated for both OS and RFS, and between-group differences were evaluated using the log-rank test. P-values less than 0.05 were considered statistically significant. All statistical analyses were performed using R version 4.4.2 (R foundation).

## Results

### Clinicopathologic patient characteristics

A total of 78 patients were included in this study, with 39 patients in each group, matched from an initial cohort of 138 patients who underwent laparoscopic gastrectomy with D2 lymphadenectomy. The clinicopathologic features of the patients are summarized in Table 1. The median age was similar between the CLI group (65.0 [58.0–76.5] years) and the ALI group (65.0 [61.0–71.0] years, p = 0.908). There were no significant differences in sex distribution (CLI vs. ALI: 61.5% male vs. 64.1% male, p > 0.999) or BMI (CLI vs. ALI: 24.3 [22.6–26.2] vs. 23.4 [21.4–25.0], p = 0.238) between the two groups. The prevalence of medical comorbidities was also similar between the groups (CLI vs. ALI: 61.5% vs. 59.0%, p = 0.817). The ASA physical status distribution showed a marginal difference between the groups, with more ASA 2 patients in the ALI group and more ASA 3 patients in the CLI group (p = 0.049).

**Table 1:** Clinicopathologic features of the conventional laparoscopic instrument (CLI) and articulating laparoscopic instrument (ALI) groups after propensity score matching

	CLI	ALI	p-value
	n = 39	n = 39	
Age (years)	65.0 (58.0–76.5)	65.0 (61.0–71.0)	0.908
Sex			> 0.999
Male	24 (61.5%)	25 (64.1%)	
Female	15 (38.5%)	14 (35.9%)	
Body mass index (kg/m^2^)	24.3 (22.6–26.2)	23.4 (21.4–25.0)	0.238
Medical comorbidity	24 (61.5%)	23 (59.0%)	0.817
ASA physical status class			0.049
1	3 (7.7%)	0 (0%)	
2	22 (56.4%)	31 (79.5%)	
3	14 (35.9%)	7 (17.9%)	
4	0 (0%)	1 (2.6%)	
TNM stage			0.553
Stage I	18 (46.2%)	17 (43.6%)	
Stage II	12 (30.8%)	9 (23.1%)	
Stage III	9 (23.1%)	13 (33.3%)	
pT stage			0.848
T1	16 (41.0%)	17 (43.6%)	
T2	3 (7.7%)	5 (12.8%)	
T3	16 (41.0%)	14 (35.9%)	
T4a	4 (10.3%)	3 (7.7%)	
pN stage			0.318
N0	23 (59.0%)	19 (48.7%)	
N1	8 (20.5%)	6 (15.4%)	
N2	6 (15.4%)	7 (17.9%)	
N3a	2 (5.1%)	7 (17.9%)	
Tumor size (mm)	35.0 (26.5–45.0)	35.0 (25.0–51.0)	0.772
Number of retrieved LN	41.0 (31.5–50.5)	42.0 (34.5–54.0)	0.535
Number of metastatic LN	0.0 (0.0–2.0)	1.0 (0.0–4.0)	0.127

Continuous data are expressed as median (interquartile range), and categorical data are expressed as n (%)

*ASA* American Society of Anesthesiologists, *TNM* tumor–node–metastasis, *pT* pathologic depth of tumor invasion, *pN* pathologic lymph node involvement, *LN* lymph nodes

Tumor characteristics, including tumor–node–metastasis (TNM) stage (p = 0.553), pT stage (p = 0.848), and pN stage (p = 0.318), were comparable between the groups. The median tumor size was the same in both groups (35.0 mm), with slightly different IQRs (CLI: [26.5–45.0]; ALI: [25.0–51.0], p = 0.772). The ALI group had slightly higher number of retrieved lymph nodes (42.0 [34.5–54.0] vs. 41.0 [31.5–50.5], p = 0.535) and metastatic lymph nodes (1.0 [0.0–4.0] vs. 0.0 [0.0–2.0], p = 0.127), although the differences were not statistically significant.

### Short-term surgical outcomes

The extent of gastrectomy was similar between the two groups (p = 0.580), with distal gastrectomy being the most common procedure (84.6% in both groups) (Table 2). The reconstruction methods also showed no significant differences (p = 0.708). Billroth II was the most frequently performed reconstruction method in both groups.

**Table 2:** Surgical outcomes of the conventional laparoscopic instrument (CLI) and articulating laparoscopic instrument (ALI) groups after propensity score matching

	CLI	ALI	p-value
	n = 39	n = 39	
Extent of gastrectomy			0.580
Distal gastrectomy	33 (84.6%)	33 (84.6%)	
Total gastrectomy	6 (15.4%)	5 (12.8%)	
Proximal gastrectomy	0 (0%)	1 (2.6%)	
Reconstruction			0.708
Billroth I	10 (25.6%)	12 (30.8%)	
Billroth II	23 (59.0%)	20 (51.3%)	
Roux-en-Y gastrojejunostomy	6 (15.4%)	6 (15.4%)	
Double-flap technique	0 (0%)	1 (2.6%)	
Operative time (min)	194.0 (151.5–238.5)	168.0 (143.5–216.5)	0.202
Estimated blood loss (mL)	50.0 (26.0–100.0)	70.0 (36.5–100.0)	0.634
Transfusion	1 (5.1%)	0 (0%)	0.152
Duration of postoperative hospitalization (days)	5.0 (4.0–7.0)	4.0 (3.0–5.0)	0.001
Time to first flatus (days)	3.0 (2.0–3.0)	2.0 (2.0–3.0)	0.004
All complications	23 (59.0%)	20 (51.3%)	0.495
Major complication (grade III or higher)	1 (2.6%)	0 (0%)	0.314

Continuous data are expressed as median (interquartile range), and categorical data are expressed as n (%)

The ALI group had a shorter operative time (168.0 [143.5–216.5] minutes vs. 194.0 [151.5–238.5] minutes) and less estimated blood loss (70.0 [36.5–100.0] mL vs. 50.0 [26.0–100.0] mL) compared with the CLI group, but these differences were not statistically significant (p = 0.202 and p = 0.634, respectively). Transfusions were required in one patient in the CLI group (5.1%), whereas none were required in the ALI group (p = 0.152).

The postoperative hospital stay was significantly shorter in the ALI group (4.0 [3.0–5.0] days vs. 5.0 [4.0–7.0] days, p = 0.001), as was the time to first flatus (2.0 [2.0–3.0] days vs. 3.0 [2.0–3.0] days, p = 0.004).

The overall complication rate was comparable between the two groups (CLI vs. ALI: 59.0% vs. 51.3%, p = 0.495). Major complications (grade III or higher) occurred in one patient (2.6%) in the CLI group, whereas none were observed in the ALI group (p = 0.314).

### Long-term oncologic outcomes

**Fig. 2:**
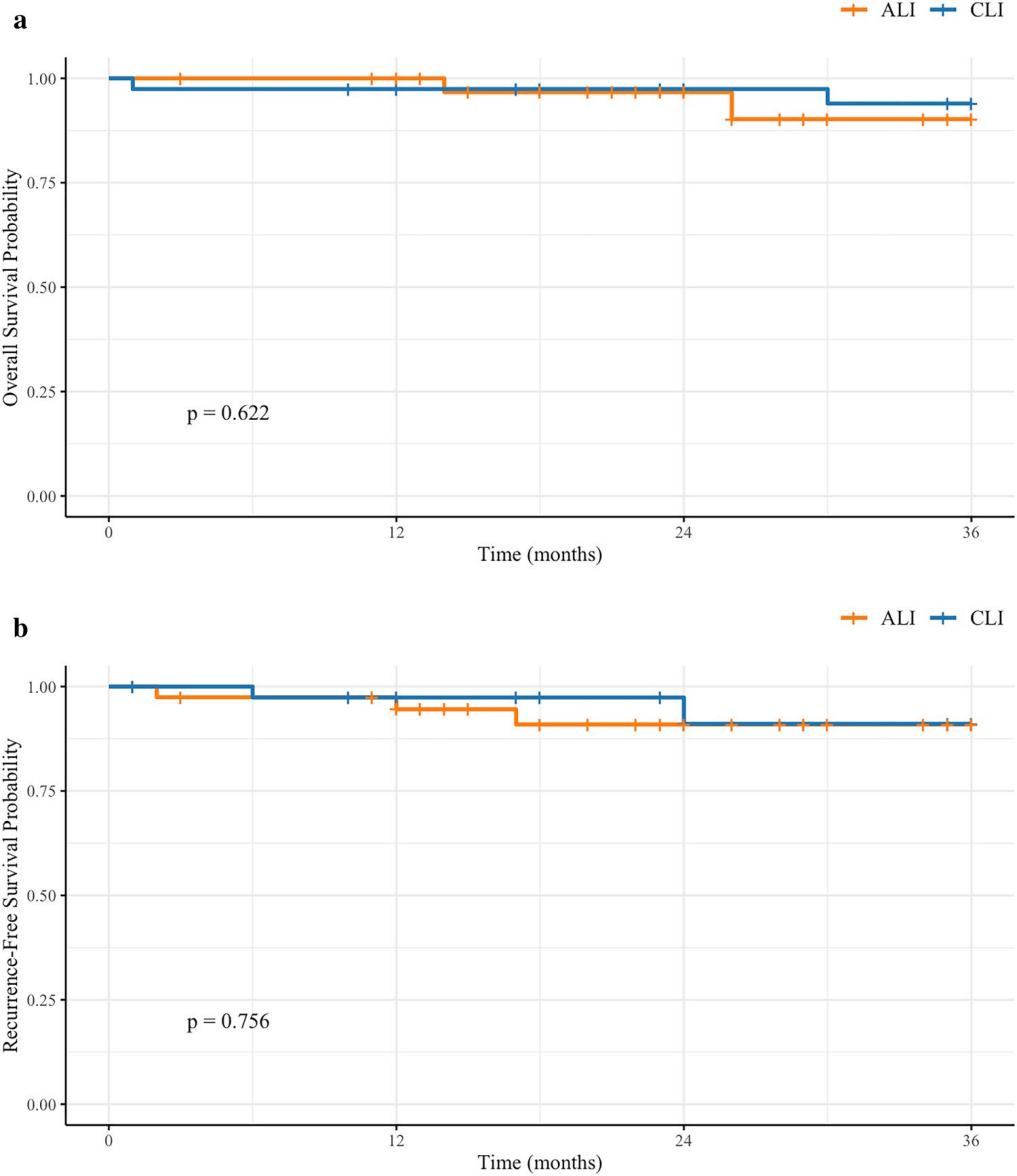
Kaplan–Meier survival curves of patients in the conventional laparoscopic instrument (CLI) and articulating laparoscopic instrument (ALI) groups. **a** Overall survival; **b** recurrence-free survival

The mean follow-up period was 26 months. During follow-up, four deaths were observed, with two occurring in the CLI group (5.1%) and two in the ALI group (5.1%). The Kaplan–Meier OS curves are shown in Fig. 2a. The log-rank test revealed no statistically significant difference in OS between the two groups (p = 0.622).

Recurrence was observed in six patients, with three cases in the CLI group and three in the ALI group. The recurrence sites included the remnant stomach, liver, peritoneum, anastomotic site, and multiple locations. The RFS curves are shown in Fig. 2b, and the log-rank test indicated no statistically significant difference in RFS between the two groups (p = 0.756).

## Discussion

In this study, compared with CLIs, ALI use was associated with improved short-term perioperative outcomes and comparable long-term oncologic outcomes in patients undergoing laparoscopic gastrectomy with D2 lymphadenectomy for gastric cancer. The ALI group demonstrated significantly faster postoperative recovery, with a shorter hospital stay and quicker time to first flatus compared with the CLI group. Operative time and estimated blood loss were also lower in the ALI group, though these differences were not statistically significant. Complication rates, including major complications, were similar between the two groups, and long-term oncologic outcomes, including OS and RFS, showed no significant differences. These findings suggest that ALIs may enhance perioperative recovery while maintaining oncologic safety in gastric cancer surgery.

The improved perioperative recovery observed in the ALI group may be attributed to the enhanced maneuverability and precision of ALIs. The instruments’ increased range of motion allows for more efficient tissue handling and precise lymphadenectomy, which may reduce surgical stress and tissue trauma. Previous studies have demonstrated that ALIs improve dexterity in confined anatomical spaces, leading to enhanced surgical efficiency and faster recovery [8, 13–15]. Specifically, the improved flexibility of ALIs facilitates precise dissection while minimizing excessive force on surrounding tissues, potentially reducing postoperative inflammation and expediting bowel function recovery. Given the importance of early recovery in postoperative outcomes, particularly in oncologic surgery, our findings suggest that ALIs may provide meaningful clinical benefits in laparoscopic gastrectomy. The faster recovery times and shorter hospital stays associated with ALIs also contribute to lower healthcare costs by minimizing hospitalization duration and resource utilization.

ALIs provided even greater advantages in more challenging surgeries in anatomically complex areas [13, 16, 17]. CLIs, with their rigid and non-articulated design, often limit the precision of dissections during D2 lymphadenectomy, particularly in hard-to-reach areas, such as No. 8a, No. 11p, and No. 12a lymph nodes. Anatomical constraints, including the convex body of the pancreas, further restrict the surgical field and hinder adequate exposure [18]. The enhanced flexibility of ALIs expands the effective operating space and enables precise dissection in narrow and deep regions. The improved accessibility of difficult-to-reach areas afforded by the greater precision and maneuverability of ALIs may reduce the risk of complications and facilitate recovery in complex cases [19, 20]. Moreover, ALIs provide a degree of freedom similar to that of robotic instruments, allowing precise manipulation in deep surgical fields where conventional instruments struggle. In a previous study comparing laparoscopic gastrectomy with ALIs and robotic gastrectomy, articulation was found to be beneficial primarily during D2 lymphadenectomy, whereas its role in reconstruction was limited [21]. The selective use of articulation contributed to reduced operation time in laparoscopic surgery with ALIs compared with robotic surgery. Based on this finding, we hypothesized that ALIs could offer specific advantages in D2 lymphadenectomy and investigated their impact in this study, ultimately confirming their benefits in this setting. As proficiency with ALIs increases, their potential benefits may extend to a broader range of procedures, including reduced-port surgery [13, 14].

This study found no significant differences in OS and RFS between the ALI and CLI groups, which suggests that ALIs allow for precise oncologic resection without compromising surgical integrity, thereby supporting their oncologic safety in gastric cancer surgery requiring D2 lymphadenectomy. Furthermore, the ALI group exhibited a slightly higher number of retrieved lymph nodes, which is a key factor influencing oncologic outcomes as adequate lymph node dissection improves staging accuracy and prognosis [22, 23]. Although traditional guidelines recommend retrieving at least 15 lymph nodes in gastric cancer, recent studies suggest that an even higher number may be beneficial, especially in advanced cases [24, 25]. As laparoscopic techniques continue to evolve, ALIs may play an increasing role in ensuring both precise lymphadenectomy and favorable oncologic outcomes in gastric cancer surgery. Further studies with larger sample sizes and longer follow-up periods are needed to validate these findings and explore whether the potential benefits of ALIs extend to long-term oncological outcomes.

This study was retrospective in nature and used propensity score matching to minimize selection bias. However, the possibility of residual confounding cannot be entirely discounted. The relatively small sample size may also limit the generalizability of the findings, particularly in detecting statistically significant differences in rare outcomes, such as major complications. This limitation may also have contributed to the lack of statistical significance in operative time, which could further be influenced by variability related to the learning curve during early ALI adoption. Another limitation of this study is the relatively short follow-up period, which may not have been adequate to fully assess long-term oncological outcomes.

In conclusion, compared with CLIs, the use of ALIs in gastrectomy with D2 lymphadenectomy was associated with improved perioperative outcomes, though no significant differences were observed in long-term oncological outcomes, such as OS and RFS. ALIs may offer distinct advantages in complex surgical procedures due to their enhanced maneuverability and precision. Further research with larger sample sizes and longer follow-up periods is necessary to fully assess the long-term oncologic impact of ALIs in gastric cancer surgery.
